# Neuroinflammation following anti-parkinsonian drugs in early Parkinson’s disease: a longitudinal PET study

**DOI:** 10.1038/s41598-024-55233-z

**Published:** 2024-02-27

**Authors:** Tatsuhiro Terada, Tomoyasu Bunai, Takanori Hashizume, Takashi Matsudaira, Masamichi Yokokura, Hirotsugu Takashima, Takashi Konishi, Tomokazu Obi, Yasuomi Ouchi

**Affiliations:** 1 Department of Biofunctional Imaging, Preeminent Medical Photonics Education and Research Center, Hamamatsu University School of Medicine, 1-20-1 Handayama, Higashi-ku, Hamamatsu, 431-3192 Japan; 2 Department of Neurology, Shizuoka Institute of Epilepsy and Neurological Disorders, Shizuoka, Japan; 3 Laboratory of Drug Metabolism and Pharmacokinetics, Faculty of Pharmacy, Osaka Ohtani University, Tondabayashi, Japan; 4 Department of Psychiatry, Hamamatsu University School of Medicine, Hamamatsu, Japan; 5 Department of Neurology, Japanese Red Cross Shizuoka Hospital, Shizuoka, Japan; 6 Hamamatsu Medical Imaging Center, Hamamatsu Medical Photonics Foundation, Hamamatsu, Japan

**Keywords:** Parkinson’s disease (PD), Neuroinflammation, Dopamine transporter, Zonisamide, Positron emission tomography, Neurology, Neurological disorders

## Abstract

The progression of neuroinflammation after anti-parkinsonian therapy on the Parkinson’s disease (PD) brain and in vivo evidence of the therapy purporting neuroprotection remain unclear. To elucidate this, we examined changes in microglial activation, nigrostriatal degeneration, and clinical symptoms longitudinally after dopamine replacement therapy in early, optimally-controlled PD patients with and without zonisamide treatment using positron emission tomography (PET). We enrolled sixteen PD patients (Hoehn and Yahr stage 1–2), and age-matched normal subjects. PD patients were randomly divided into two groups: one (zonisamide^+^) that did and one (zonisamide^−^) that did not undergo zonisamide therapy. Annual changes in neuroinflammation ([^11^C]DPA713 PET), dopamine transporter availability ([^11^C]CFT PET) and clinical severity were examined. Voxelwise differentiations in the binding of [^11^C]DPA713 (BP_ND_) and [^11^C]CFT (SUVR) were compared with normal data and between the zonisamide^+^ and zonisamide^−^ PD groups. The cerebral [^11^C]DPA713 BP_ND_ increased with time predominantly over the parieto-occipital region in PD patients. Comparison of the zonisamide^+^ group with the zonisamide^−^ group showed lower levels in the cerebral [^11^C]DPA713 BP_ND_ in the zonisamide^+^ group. While the striatal [^11^C]CFT SUVR decreased longitudinally, the [^11^C]CFT SUVR in the nucleus accumbens showed a higher binding in the zonisamide^+^ group. A significant annual increase in attention score were found in the zonisamide^+^ group. The current results indicate neuroinflammation proceeds to the whole brain even after anti-parkinsonian therapy, but zonisamide coadministration might have the potential to ameliorate proinflammatory responses, exerting a neuroprotective effect in more damaged nigrostriatal regions with enhanced attention in PD.

## Introduction

Parkinson’s disease (PD) is a neurodegenerative disorder associated with the loss of dopaminergic neurons in the substantia nigra and the accumulation of the aggregated form of the α-synuclein protein^1^. In addition to motor dysfunction, PD patients suffer nonmotor impairments, including cognitive decline, even at the early disease stage, indicating that not only the nigrostriatal system but also cortical involvement are implicated in its pathophysiology^2^. In addition to α-synuclein pathology, neuroinflammation is considered to be an important contributor to disease onset and progression^3^. Although the precise role of neuroinflammation (different polarization of microglia) in PD is currently unclear, recent studies have indicated that α-synuclein aggregation activates microglia that proceed over cerebral cortical regions during the early-stage period in PD^3,4^.

From the therapeutic point of view, although dopamine replacement therapy improves the motor symptoms and exerts some neuroprotective effects^5^, it is unclear whether such anti-parkinsonian drugs continue to suppress microglial activation even under the proper intervention for the motor impairments. Among some anti-inflammatory agents acting as neuroprotective drugs for PD, zonisamide was reported to have a beneficial effect on motor improvement in PD patients^6^. Recent animal experiments revealed that zonisamide acts on neuroinflammatory glial cells, resulting in neuroinflammation suppression as a neuroprotective agent in PD^7^. Additional neuroprotective effects have been reported with zonisamide, including inhibition of microglial activation^7–9^. However, this promising effect of zonisamide has not been replicated in the living human brains of PD patients.

The 18-kDa translocator protein (TSPO) on the outer membrane of mitochondria is highly expressed in activated microglia, which makes TSPO a marker of neuroinflammation in the brain^10^. Positron emission tomography (PET) specifically delineates the TSPO density in activated microglia. A second-generation PET tracer, [^11^C]DPA713, has been reported to have a higher affinity for TSPO^10^. The dopamine transporter in the presynaptic dopaminergic terminal regulates the synaptic concentration of dopamine. [^11^C]CFT is a PET ligand for dopamine transporter in the presynaptic dopaminergic terminal, reflecting the degeneration of nigrostriatal neurons in PD^11^.

The aim of this study was to investigate the progression of microglial activation in early-stage PD patients under the proper anti-parkinsonian drug intervention using PET in a longitudinal study, in which four examinations per patient of the level of microglial activation by [^11^C]DPA713, the degree of nigrostriatal dopamine degeneration evaluated by [^11^C]CFT and cognition and behavior by multiple cognitive-behavioral tests were performed in PD patients treated optimally with dopamine replacement therapy with and without zonisamide.

## Materials and methods

### Study design

This longitudinal study comprised 2 stages: the observation phase before imaging and treatment (baseline evaluation) and the treatment phase (3 years). From August 2015 to September 2017, we enrolled stable patients with early-stage PD on levodopa/dopa decarboxylase inhibitor (DCI) therapy. Patients diagnosed with definite PD based on the clinical criteria of the United Kingdom PD Brain Bank^12^ were eligible for inclusion in this study. All patients were recruited from our hospital or neighboring 6 hospitals. Major inclusion criteria were as follows: patients whose disease severity was at an early stage based on the Hoehn and Yahr stage at 1 or 2; patients on early stage of PD receiving levodopa/DCI with a history of treatment with antiparkinsonian drugs other than levodopa/DCI and zonisamide; patients aged under 80 years; patients who voluntarily provided written informed consent; and patients who were able to complete the longitudinal follow up at least 1 year in this study. Major exclusion criteria were the followings: patients with Parkinson syndrome other than PD; patients with epilepsy; patients who received any surgery for PD within 6 months before screening; patients who received zonisamide, selegiline and/or pramipexole within 3 months before screening because previous reports have shown neuroprotective effects of these drugs^13,14^; patients with any severe psychiatric symptoms, such as confusion, hallucination, delusion and abnormal behaviors; patients with any histories of malignant syndrome; and patients with a history of drug allergy for zonisamide. In addition, participants with cerebrovascular disease, hydrocephalus, brain tumor, epileptic foci, or traumatic brain injury were excluded based on brain magnetic resonance imaging (MRI) findings. Subjects who met the criteria of dementia with Lewy bodies were also excluded^15^. In the course of the study, patients who developed symptoms that met the criteria for the diagnosis of probable progressive supranuclear palsy^16^, corticobasal degeneration^17^, and multiple system atrophy were excluded^18^ as well. To confirm the biological diagnosis of PD, a dopamine transporter PET scan with [^11^C]CFT was performed at the beginning of this study. We confirmed that all patients showed a milder to moderate reduction in [^11^C]CFT uptake in the striatum (dominantly in the posterodorsal putamen). The clinical severity of parkinsonism in all patients corresponded to either Hoehn and Yahr stage 1 or 2, indicating early-stage PD.

**Figure 1:**
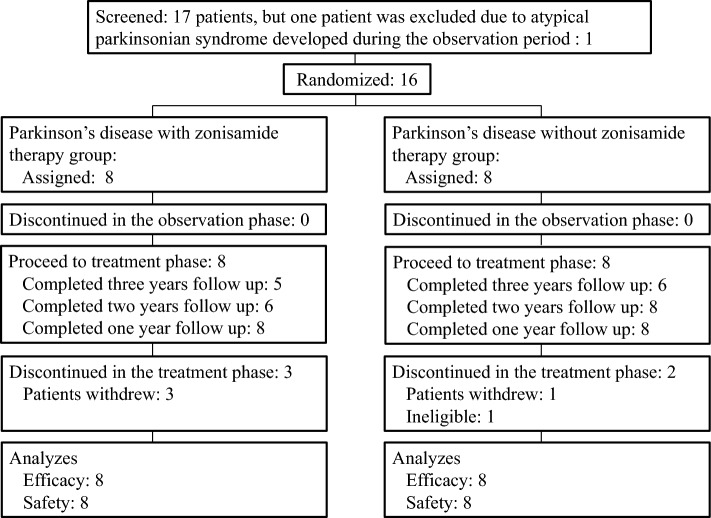
Patient disposition. All 16 PD patients were scanned with two PET tracers and evaluated clinically. After the first (baseline) scan and clinical assessment, a fixed dose of levodopa/dopa decarboxylase inhibitor (DCI) plus zonisamide at 25 mg/day was given in the zonisamide^+^ group, while the same anti-parkinsonian drugs used during the observation period were continued in the zonisamide^−^ group for at least 1 year. During the treatment phase, clinical assessment, a pair of PET scans, and a measure of plasma concentration of zonisamide were completed at every visit (3 times per patient).

During the 1-month observation phase, a fixed dose of levodopa/DCI, was required for all patients. All patients were evaluated neurologically and with clinical batteries, including the Unified PD Rating Scale (UPDRS) for parkinsonism^19^ and PD Questionnaire-39 (PDQ-39) summary index for quality of life^20^, Mini-Mental State Examination (MMSE) for global cognitive function^21^ and neuropsychiatric Inventory (NPI) for neuropsychiatric symptoms^22^, before and after this follow-up study. After the observation phase, sixteen patients were randomly categorized into two groups: one with zonisamide treatment (zonisamide^+^) (n = 8) and the one without zonisamide treatment (zonisamide^−^) (n = 8) (Fig. 1). During the treatment phase, clinical assessment, a pair of PET scans, and a measure of plasma concentration of zonisamide were completed at every visit (3 times per patient). In addition, safety issues, such as the incidence of adverse events, vital signs, and general laboratory tests, were examined at every visit. After a 1-year fixed dose period, the following treatments were allowed: (1) zonisamide 50 mg/day only in the zonisamide^+^ group, (2) additional administration of levodopa/DCI, and (3) additional administration of dopamine agonists according to symptoms varying during the following 2-year-long period. One patient in the zonisamide^−^ group received amantadine from the observation period.

A total of twenty-four age-matched normal control subjects were also recruited by in-house advertisements. The control group for the [^11^C] *N*,*N*-diethyl-2-[2-(4-methoxyphenyl)-5,7-dimethylpyrazolo[1,5-a]pyrimidin-3-yl]acetamide (DPA713) evaluation consisted of 15 healthy subjects (5 men and 10 women: mean age ± SD, 71.1 ± 7.3 years, all right handed), and the control group for the [^11^C] 2β-carbomethoxy-3β-(4-fluorophenyl)tropane (CFT) SUVR evaluation consisted of 9 healthy subjects (4 men and 5 women: mean age ± SD, 62.9 ± 6.8 years, all right handed). All healthy controls had no history of head injury, psychiatric disease, serious medical illness or surgery. In addition, they had no neurological problems, and their Clinical Dementia Rating was zero, indicating no dementia^23^. The exclusion criteria of MRI findings described in “Materials and methods” (study design section) were also applied to the controls. No significant differences in age or sex were found between the two control groups.

This study was reviewed and approved by the Clinical Research Review Board of Hamamatsu University School of Medicine (jRCTs041180070) in accordance with the Declaration of Helsinki. Written informed consent was obtained from all participants.

### MRI scanning

Before the series of PET scans, 3.0 Tesla magnetic resonance imaging (MRI) (Ingenia 3.0 T, PHILIPS) of the whole brain was performed on all participants in 3-dimensional mode (Supplementary methods).

### PET measurement

All participants underwent a series of PET measurements using a high-resolution brain PET scanner (SHR12000; Hamamatsu Photonics K.K., Hamamatsu, Japan). Dynamic PET scans were performed after intravenous injections of [^11^C]DPA713 and [^11^C]CFT. For all patients, the PET measurements of [^11^C]DPA713 and [^11^C]CFT were performed annually for 3 years (four times per patient) (Supplementary methods).

### PET imaging data processing

All PET data processing procedures were performed using PMOD 3.4 software (PMOD Technologies Ltd., Zurich, Switzerland). To assess microglial activation in the brain, the binding potential (BP_ND_) of [^11^C]DPA713 was estimated with the simplified reference tissue model (SRTM) as described previously^4^ (Supplementary methods).

To assess the dopamine transporter density, we used a conventional method of the standardized uptake value ratio (SUVR) because the value of this uptake ratio reflects the BP_ND_ as reported previously^11,24^ (Supplementary methods).

Symptom-based hemispheric differences in PD patients were effaced by flipping PET images. The left side of the images was set as contralateral to the side of more severe parkinsonism clinically, showing more affected [^11^C]CFT binding in the striatum.

All PET images were normalized to Montreal Neurological Institute space and smoothed with an isotropic Gaussian kernel of 6 mm at FWHM by using Statistical Parametric Mapping 12 software (SPM12; Wellcome Department of Imaging Neurosciences, London, UK; http://www.fil.ion.ucl.ac.uk/spm) running on MATLAB 7.12.0 (The MathWorks, Natick, MA, USA)^24^.

### Voxel-based analysis and statistics

We performed a voxel-based analysis of the whole brain using SPM12. To identify the region that exhibited increased levels of [^11^C]DPA713 BP_ND_ and decreased levels of [^11^C]CFT SUVR in the patients with PD compared with controls, group comparison was performed with a two-sample *t* test in SPM12 using age and sex as covariates of no interest. The validity of the procedure using parametric PET images has been recognized by previous reports^4,10^.

To investigate the longitudinal changes of the [^11^C]DPA713 BP_ND_ and [^11^C]CFT SUVR in PD patients with or without zonisamide therapy, paired *t* tests in SPM12 were performed between the PD groups for the first PET scan and second, third or fourth PET scan.

In [^11^C]DPA713 PET analysis, a voxel-level height threshold was set at p < 0.001 (uncorrected for multiple comparisons) without the extent threshold, because the test was exploratory in nature with a priori knowledge of microglial activation. Furthermore, the purpose of this analysis was to characterize the progression pattern in an exploratory way. Indeed, the 1-year interval between PET scans might be relatively short. To emphasize the different patterns of progression in [^11^C]DPA713 binding in group comparison, we used a voxel-level height threshold set at p < 0.00001.

In [^11^C]CFT PET analysis, a voxel-level height threshold was set at p < 0.05 corrected for multiple comparisons using family-wise error (FWE).

In addition, we determined whether there were differences in baseline (initial PET scan) [^11^C]DPA713 BP_ND_ levels with or without zonisamide therapy at either level p < 0.05 (FWE) or voxel-level height threshold p < 0.001 (uncorrected for multiple comparison).

### ROI analysis and statistics

Manual ROIs were traced on the individual MRI scans, not on normalized MRI scans, with PMOD software. ROIs were manually located bilaterally on the thalamus, caudate, putamen, nucleus accumbens, precuneus, temporal, parietal, occipital and middle frontal cortex, pons, and midbrain according to the atlas (Supplementary Fig. 1). These ROIs were automatically transferred onto corresponding individual [^11^C]DPA713 BP_ND_ or [^11^C]CFT SUVR parametric images. The regional [^11^C]DPA713 BP_ND_ level or [^11^C]CFT SUVR level was determined from these manual ROIs. The data were analyzed by using the Statistical Package for Social Sciences version 19 (SPSS Inc, Chicago, IL, USA).

To investigate the difference in longitudinal changes in the regional [^11^C]DPA713 BP_ND_ or [^11^C]CFT SUVR from baseline (first PET scan) to that observed 3 years later (fourth PET scan) between the two PD groups with or without zonisamide, we applied repeated two-way analysis of variance (ANOVA). Statistical significance was set at p < 0.05.

To confirm the consistency of data from the manual ROI analysis, an automated ROI analysis was also performed. ROIs were created using WFU PickAtlas, which provides ROI masks based on Talairach Daemon (TD) labels or automatic anatomic labeling (aal). These ROIs were automatically transferred onto corresponding normalized and smoothed [^11^C]DPA713 BP_ND_ or [^11^C]CFT SUVR parametric images. Then, regional PET data were determined, and statistical analysis was performed.

### Analysis of zonisamide in plasma

The plasma concentration of zonisamide was annually analyzed using a HPLC–UV method (Supplementary methods).

### Statistical analysis of clinical scores

The difference in the longitudinal changes in clinical scores from baseline (first PET scan) to 3 years later (fourth PET scan) between the zonisamide^+^ and zonisamide^−^ groups was evaluated by repeated two-way ANOVA using SPSS. Normal distribution was examined by Levene’s test in SPSS. Statistical significance was set at p < 0.05.

### Data sharing

Anonymized data are available on request to the corresponding author.

## Results

### Study participants and demographic or clinical characteristics

Of the 17 patients screened, one patient was excluded due to atypical parkinsonian syndrome that developed during the observation period. Among 16 patients with PD recruited at entry, five participants found it difficult to visit regularly (Fig. 1). The reasons for discontinuation were adverse events directly irrelevant to this study (1 patient), patient withdrawal (4 patients), inaccessibility (1 patient). No significant differences in clinical evaluations or laboratory tests including age, disease duration, parkinsonism, cognitive function, and medication were found between the zonisamide^+^ and zonisamide^−^ groups (Table 1). There were no significant age or gender differences between the PD group and normal control subjects both in [^11^C]DPA713 (*t*-test, p > 0.05) and [^11^C]CFT (*t*-test, p > 0.05) assessments.

**Table 1:** Demographic and clinical characteristics of the Parkinson’s disease (PD) group at baseline.

	PD group			*P* value
	Total (N = 16)	Subgroup with zonisamide treatment (N = 8)	Subgroup without zonisamide treatment (N = 8)	
Age (years)	65.6 ± 9.2	64.5 ± 12.7	66.6 ± 4.0	n.s.
Men/women (number)	9/7	4/4	5/3	n.s.
Disease duration (years)	2.8 ± 2.2	3.3 ± 2.4	2.4 ± 1.9	n.s.
Affected side (right/left)	7/9	3/5	4/4	n.s.
Hoehn and Yahr (I/II)	2/14	0/8	2/6	n.s.
Unified PD Rating Scale I (/16)	1.9 ± 1.5	2.4 ± 1.1	1.4 ± 1.7	n.s.
Unified PD Rating Scale II (/52)	6.5 ± 2.3	7.4 ± 2.7	5.6 ± 1.7	n.s.
Unified PD Rating Scale III (/108)	16.4 ± 7.4	17.1 ± 5.3	15.6 ± 9.5	n.s.
Mini-Mental State Examination (/30)	26.7 ± 2.2	26.1 ± 2.0	27.3 ± 2.5	n.s.
Neuropsychiatric Inventory (/144)	3.3 ± 4.1	3.8 ± 2.7	2.9 ± 5.4	n.s.
PD Questionnaire-39 summary index (/100)	11.1 ± 8.1	11.5 ± 6.1	10.8 ± 10.1	n.s.
Levodopa daily dose (mg/day)	334.4 ± 166.1	331.3 ± 146.2	337.5 ± 194.1	n.s.
Levodopa equivalent daily dose (mg/day)	337.5 ± 161.8	331.3 ± 146.2	343.8 ± 186.0	n.s.

Data are presented as the mean ± SD (range).

One patient in subgroup without zonizamide had taken amantadine at entry (50 mg/day). Conversion factor for the levodopa equivalent daily dose: amantadine, × 1.

### Longitudinal changes in [^11^C]DPA713 BP_ND_ in PD as a whole

**Figure 2:**
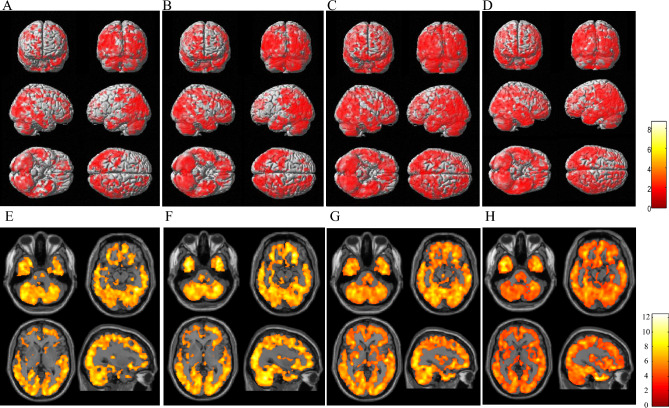
Regions of the significant increase in [^11^C]DPA713 BP_ND_ in the PD group compared with normal controls. (**A**,**E**) The first (baseline) scan. (**B**,**F**) The second (1 year later) scan. (**C**,**G**) The third (2 years later) scan. (**D**,**H**) The fourth (3 years later) scan. The results are displayed as three dimensionally rendered brain-surface images at a voxel-level height threshold of p < 0.00001 (uncorrected for multiple comparisons) (upper) (**A**–**D**) and canonical T1 images at a voxel-level height threshold of p < 0.001 (uncorrected for multiple comparisons) (lower) (**E**–**H**). The color bar represents the T value. SPM analysis showed a significantly increased [^11^C]DPA713 BP_ND_ in the bilateral parieto-occipital cortex, including the caudate and brainstem, in the total PD group in the first PET scan compared to the normal control group (**A**,**E**). Significant increases in [^11^C]DPA713 BP_ND_ in the second, third, and fourth scans were found in PD patients, spreading more broadly to the whole brain (**B–D**, **F–H**).

Regardless of medical treatment, the level of [^11^C]DPA713 BP_ND_ was significantly higher in the whole brains of patients in the PD group than in those of patients in the normal control group, and the increase in its binding became more evident, especially over the bilateral parieto-occipital and temporal cortices, as time advanced (Fig. 2).

In addition, we found no significant difference in [^11^C]DPA713 BP_ND_ levels between the zonisamide^+^ and zonisamide^−^ groups at baseline (p < 0.001, uncorrected and p < 0.05, FWE).

### Longitudinal changes in [^11^C]DPA713 BP_ND_ in both PD groups

**Figure 3:**
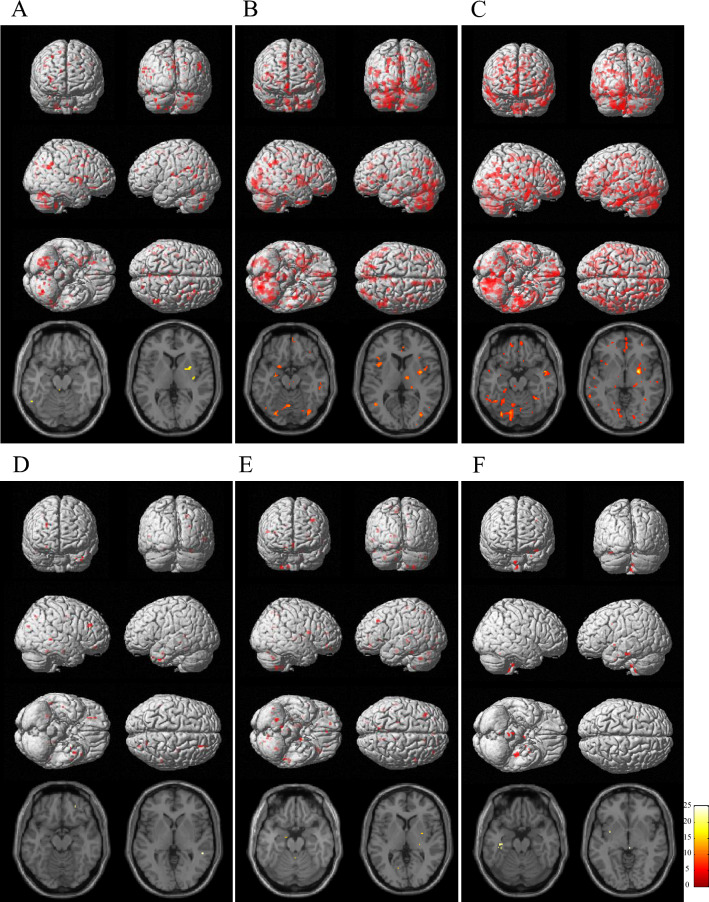
Longitudinal changes in [^11^C]DPA713 BP_ND_ in PD patients. (**A**–**C**) The zonisamide^−^ group. (**D**–**F**) The zonisamide^+^ group. Compared with data in the first scan, regions with significant increases in [^11^C]DPA713 BP_ND_ are shown in the second (**A**,**D**), third (**B**,**E**) and fourth (**C**,**F**) scans. The results are displayed as three-dimensionally rendered brain-surface images (upper) and canonical T1 images (lower). A voxel-level height threshold was set at p < 0.001 (uncorrected for multiple comparisons). The color bar represents the T value. In the zonisamide^−^ group, several clusters with diffuse and patchy appearance were identified in the second scan (**A**), and the regions with a significant increase gradually widened in the following scans (**B**,**C**). In contrast, in the zonisamide^+^ group, significant increases in the second, third and fourth scans were rather limited and small (**D**–**F**).

In the zonisamide^+^ group, a significant increase in [^11^C]DPA713 BP_ND_ was observed in rather limited brain areas, the annual increase of which was apparently smaller than that in the zonisamide^−^ group (Fig. 3).

**Figure 4:**
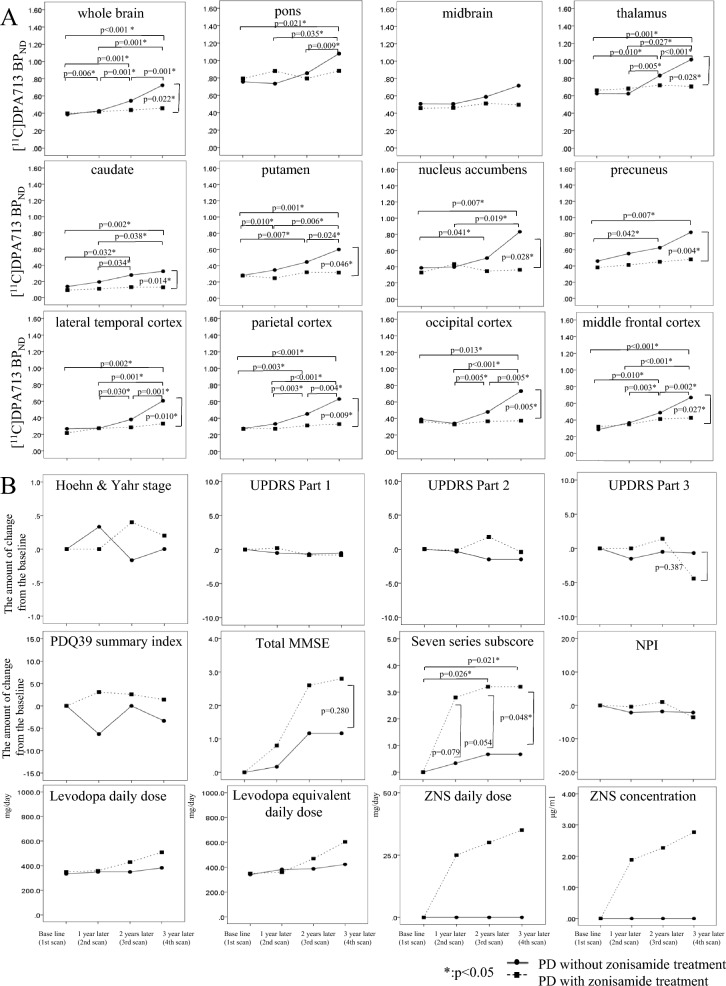
Longitudinal changes in the PD related data in both PD groups. (**A**) Longitudinal changes in the [^11^C]DPA713 BP_ND_ in both PD groups based on ROI analyses. There was no significant difference in the [^11^C]DPA713 BP_ND_ between groups in the first scan (baseline). In the zonisamide^−^ group, significant [^11^C]DPA713 BP_ND_ increases were found in all ROIs (pons, thalamus, caudate, putamen, nucleus accambens, precuneus, temporal cortex, parietal cortex, occipital cortex, and middle frontal cortex) but not in the zonisamide^+^ group. These results were consistent with those from the voxelwise analyses, as shown in Fig. 2. (**B**) Longitudinal changes in the clinical scores in both PD groups. Among scores of UPDRS, PDQ-39 summary index, MMSE, and NPI, the seven series subtest showed significantly higher score at the fourth visit in the zonisamide^+^ group than in the zonisamide^−^ group. No significant longitudinal changes in seven series subscore were seen in the zonisamide^−^ group. Regarding medication, while small increases in the doses of levodopa and zonisamide were seen at a later visit, there was no significant difference in such doses between groups.

Consistently, the manual ROI analysis showed a longitudinally remarkable increase in [^11^C]DPA713 BP_ND_ from baseline to that observed 3 years later in the cerebral cortex, striatum, and thalamus in the zonisamide^−^ group but a mild increase in the zonisamide^+^ group (Fig. 4A) (Supplementary Table 1A). However, no significant increase was observed from baseline to 1 or 2 years later.

The results of the automated ROI analysis were similar to those of the manual ROI analysis (Supplementary Fig. 2; Supplementary Table 1B).

### Longitudinal changes in clinical scores in both PD groups

No significant longitudinal progression in the score of motor symptoms (Hoehn and Yahr stage, UPDRS, and PDQ-39 summary index) were observed in PD group, suggesting our PD patients were well controlled and stable (Fig. 4B) (Supplementary Table 1C).

A significant increase in changes in the seven series subtest of the MMSE from baseline was observed in the zonisamide^+^ group, whereas there was no significant change in the zonisamide^−^ group (Fig. 4B). In addition, the changes in the 7-series subtests after 3 years in the zonisamide^+^ group were significantly higher than in the zonisamide^+^ group, but there were no significant differences in the temporal changes from baseline to 1 or 2 years between groups (Fig. 4B) (Supplementary Table 1C). All other assessments exhibited no significant changes between groups over time.

Regarding medication, two patients were treated with 50 mg/day of zonisamide after the first 1-year fixed medication period, and a small amount of levodopa/DCI and, in addition, other anti-parkinsonian drugs were administered to a few patients (Supplementary Table 2).

The longitudinal changes from the baseline of the UPDRS, Hoehn and Yahr stage, PDQ-39 summary index, NPI, levodopa/DCI daily dose or levodopa equivalent daily dose showed no significant difference between groups (Fig. 4B) (Supplementary Table 1C).

### Longitudinal changes in [^11^C]CFT SUVR in PD patients

**Figure 5:**
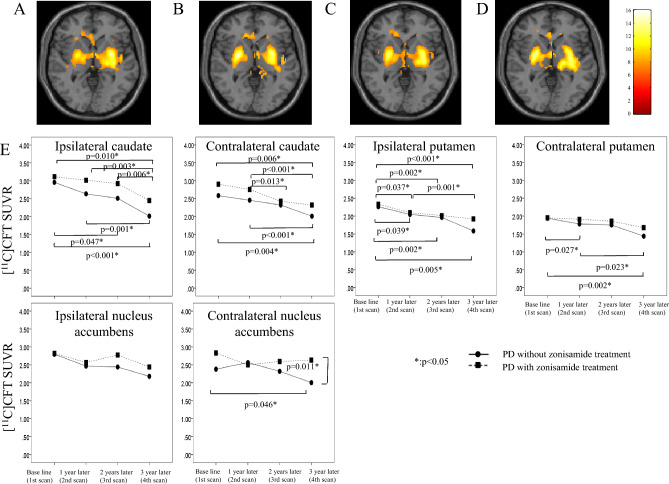
Longitudinal changes of [^11^C]CFT SUVR in both PD groups. SPM analyses showed regions of significant decreases in [^11^C]CFT SUVR in the total PD group compared with that in normal controls in the first (**A**), second (**B**), third (**C**) and fourth (**D**) scans. The results are displayed on canonical T1 images. A voxel-level height threshold was set at p < 0.05 (Family-wise error (FWE) corrected for multiple comparison). The color bar represents the T value. The left putamen contralateral to the parkinsonism shows significant reduction in [^11^C]CFT SUVR (**A**), followed by bilateral reduction in the putamen in a progressive manner (**B**–**D**). No significant longitudinal changes in [^11^C]CFT SUVR in the caudate and putamen were observed between groups (**E**). In the zonisamide^−^ group, significant [^11^C]CFT SUVR decrease were found in the contralateral nucleus accumbens but not in the zonisamide^+^ group (**E**). The [^11^C]CFT SUVR showed significantly higher at the fourth scan in the zonisamide^+^ group than in the zonisamide^−^ group (**E**).

Regardless of treatment, at the first PET scan, the PD group showed a significant decrease in [^11^C]CFT SUVR in the bilateral striatum, especially in the left (more affected) putamen, compared with controls (Fig. 4A). The decrease intensified with time, and the phenomenon was clearly seen in the contralateral (right) putamen after a 1-year period (Fig. 5A–D).

Group comparison using manual ROI analysis showed no significant difference in the longitudinal changes in the [^11^C]CFT SUVR between the zonisamide^+^ and zonisamide^−^ groups in the caudate and putamen, while the slope of the change within 2 years was somewhat milder in the affected putamen in the zonisamide^+^ group (Fig. 4E). While a unidirectional reduction in [^11^C]CFT SUVR was found in the contralateral (left) nucleus accumbens in the zonisamide^−^ group, the zonisamide^+^ group showed the higher level of [^11^C]CFT SUVR at the fourth PET scan (3 years later) compared to the zonisamide^−^ groups (Fig. 5E) (Supplementary Table 1D). Similar results were replicated in the automated ROI analysis (Supplementary Fig. 3; Supplementary Table 1E).

## Discussion

The present study is the first to show the wide spread progression of the activated microglia after the anti-parkinsonian therapy, and in vivo anti-inflammatory effect of zonisamide evidenced by the resultant reduction in the level of [^11^C]DPA713 BP_ND_ in the entire PD brain. This study demonstrated a significant elevation in the level of [^11^C]DPA713 BP_ND_ that appeared progressively over time in the whole brain, including the brainstem and striatum, in the therapeutically stable PD patients first rated at Hoehn and Yahr stages 1 and 2; this finding indicated that activated microglia spread over the extrastriatal regions even at the early stage of PD whose motor symptoms were well controlled by mainly levodopa/DCI. In contrast, the longitudinal PET measurements showed an elevation of [^11^C]DPA713 BP_ND_ reducing progressively in the cortical area, striatum and thalamus 3 years after zonisamide coadministration, suggesting the potential therapeutic capability of zonisamide to reduce proinflammatory responses developed in the brains of PD patients. Furthermore, the suppression of [^11^C]CFT binding decline in the nucleus accumbens after zonisamide therapy supports the notion that zonisamide has a protective effect on the presynaptic dopaminergic terminals in the ventral nigrostriatal region which is functionally important in attentional cognition^25,26^. Compatibly, the increased neuropsychological score of the seven series after zonisamide coadministration suggested that zonisamide can improve the aspects of attention that are affected early in PD^2,27^. Taken together, although the neuroinflammation proceeds in the entire brain of clinically stable, early PD patients even after the dopamine replacement therapy, we confirmed that zonisamide coadministration is beneficial in a clinical setting in that it exerts a neuroprotective effect on inflammatory glial cells and dopamine neurons, allowing to keep patients attentionally active.

Although the presence of microglial activation in the PD brain through postmortem and in vivo molecular imaging studies receives general acceptance among researchers, it is increasingly recognized that microglial activation occurs earlier in the PD spectrum (synucleinopathy)^3^. We found the extrastriatal spreading of the microglia activation, as shown in this study and reported elsewhere^3,4^. With time, such activation proceeds toward the cerebral cortex, including the parieto-occipital cortex even under the proper drug intervention for motor impairments. Previous studies reported that α-synuclein aggregation activates microglia by proinflammatory molecules, and in turn, once activated, microglia accelerate the propagation of α-synuclein to aggregate through the ensuing inflammatory processes^3,28^. This vicious cycle of neuroinflammation and α-synuclein propagation/aggregation might contribute to the prolonged progression of PD pathology^3^. It has been reported that α-synuclein in the brain initially aggregates in the olfactory nucleus and brainstem, followed by the substantia nigra and limbic and cortical areas based on the spread hypothesis^1,29^. As seen in Fig. 2, which shows a moderate increase in [^11^C]DPA713 binding in the olfactory region as well, it is conceivable that an enduring spread pattern of activated microglia in this study might reflect the intracerebral distribution of α-synuclein accumulation. On the other hand, these activated microglia seem to progress even under the medication of mainly levodopa/DCI. While previous in vitro studies indicated that levodopa acts as neuroprotective agent, several reports suggested that the high dose and long-term levodopa therapy might be harmful by a mechanism that probably involves oxidative stress^30^. In this study, our PD patients are at the early-stage and their motor symptom were well controlled under the therapy with mild dose of levodopa/DCI and/or small amount of amantadine. Mounting evidence from bench experiments shows that zonisamide has a neuroprotective effect across multiple animal models of PD, in which zonisamide attenuates α-synuclein-induced neurotoxicity in a manner that is independent of α-synuclein aggregates^31^, scavenges free radicals^32^, inhibits mitochondrial reactive oxygen species generation^8,33^, and inhibits microglial activation^8,9,34^. Our in vivo imaging study from a clinical point of view was able to add a significant piece of evidence that zonisamide works beneficially in the brains negatively affected by PD by suppressing activated microglia, possibly through the pharmacological actions shown in the bench.

Our longitudinal study showed that zonisamide coadministration improved attention measured by the seven series of the MMSE. Psychopathophysiologically, frontal dysfunction, such as executive dysfunction and attention, has been regarded as a primary cognitive impairment developed in the early stage of PD^2,27^. Dopamine depletion reportedly impairs frontal function by affecting the fronto-striatal thalamic circuit^35^, within which both α-synuclein-derived Lewy bodies and activated microglia are concurrently observed^3,29^. Under these circumstances, zonisamide would be able to suppress the activated microglia in the brain regions covering the frontal cortex, striatum, and thalamus, as shown in Fig. 3. The longitudinal changes in the suppression of microglial activation and attentional improvement under zonisamide treatment in the current study suggest that activated microglia (the bad guys) may play a key role in the worsening of attentional function independent of dopaminergic contribution. Despite a different study setting, suppression of microglia with minocycline would favor the decision-making process in humans^36^. Hence, microglial activation itself can be a cause of altering the normal cognitive network, specifically attention. In addition, this longitudinal study showed that the total score of the MMSE tended to increase after 3 years. The MMSE measures global cognitive function and consists of eleven items that assess various cognitive domains such as orientation, memory, attention, language, and construct ability^21^. Therefore, our study suggested that the main factor for the increasing trend in MMSE total scores was improved attention. On the other hand, although there was no significant difference after 3 years, total MMSE scores seemed to tend to increase in both PD groups. Several studies have shown the beneficial effects of levodopa on executive dysfunction associated with dopamine depletion^37,38^. Appropriate use of dopamine agonists may have influenced MMSE performance in both PD groups. A previous clinical trial (3 months long) with incremental administration of zonisamide up to 100 mg/day reported the effect of zonisamide in improving motor impairments by increasing dopamine synthesis in PD^39^. A similar clinical trial (3 months duration) on dementia with Lewy disease (DLB) revealed a positive effect of zonisamide on the improvement of parkinsonism and cognitive impairments^40^. In contrast, the lack of a significant difference in motor recovery between the PD groups with and without zonisamide in the present 3-year follow-up study might be partly due to the low dose of zonisamide administered throughout the study, where only two patients received 50 mg/day of zonisamide after 1 or 2 years, or proper intervention of the other anti-parkinsonian drugs for each patient condition clinically. This was consistent with the lack of difference in [^11^C]CFT uptake between groups. In addition, no difference in levodopa equivalent daily dose between groups in the present study indicated that anti-parkinsonian drugs other than zonisamide were unlikely to be a significant confounding factor.

In the present study, the reduced [^11^C]CFT uptake reflecting the loss of integrity of the dopamine transporter was predominantly in the putamen contralateral to the clinically affected side, indicating that our PD patients in general were at an early disease stage^41^. As the disease progresses, damage to the striatal region appears on both sides, as shown in Fig. 5. However, zonisamide mitigated the demise of dopamine terminals in the nucleus accumbens as seen in Fig. 5E. Although zonisamide’s pharmacological profile is complicated as described above, zonisamide was reportedly found to attenuate the loss of dopaminergic neurons in animal studies^31,42^, and enhance dopamine synthesis^43^. A positive effect of zonisamide on the dopamine transporter was also reported in the previous 1-year follow-up study with single photon emission computed tomography by using ^123^I-FP-CIT^44^. A close look at our longitudinal PET study focusing the striatum and accumbens showed that zonisamide coadministration reduced the level of activated microglia, which are associated with the continuing loss of dopaminergic neurons^8^ (Fig. 4A). Therefore, these results indicated that the neuroprotective mechanism of zonisamide against the presynaptic dopaminergic degeneration might reside in suppression of microglial activation. The nucleus accumbens is a ventral part of the striatum and modulator of the reward network through its projection to the frontal cortex. It therefore plays an important role in executive and emotional functions^45^. The nucleus accumbens atrophy is an established characteristic of the early stage PD and is correlated with cognitive and behavioral symptoms such as apathy in PD^45^. Apathy, one of predominant behavioral alternations seen in PD patients, is a syndrome with functionally and anatomically heterogeneous etiology mediated from the frontal area and is associated with several cognitive dysfunctions such as attention and executive dysfunction^2^. The slowing of dopamine transporter reduction in the nucleus accumbens after zonisamide treatment might at least partially explain the attentional improvement found in this study. However, further study is needed to confirm this assumption. It suffices the statistics.

There were several limitations in this longitudinal study. Due to the strict inclusion and exclusion criteria, the sample size is relatively small in terms of a cross-sectional context. However, since the main purpose of this study was to follow the same patients longitudinally for more than 3 years, the number of observations suffices the current statistical analyses. Despite this, in this study aiming at PD at an early stage, the possibility of the anti-inflammatory effect of zonisamide in a clinical setting could not be applied to PD patients at an advanced stage. Further studies with a larger sample size covering all stages of PD are needed to confirm the anti-neuroinflammatory effect of zonisamide in this disease.

In conclusion, although neuroinflammation already exists even at an early stage of PD, neuroinflammation spreads to the whole brain even under the proper intervention for the motor impairments after dopamine replacement therapy. In this process, zonisamide coadministration might help reduce neuroinflammation entirely in the brain and mitigate the dopaminergic terminal demise in the ventral nigrostriatal region. This medication also improves impaired attention. Therefore, as evidenced experimentally, this longitudinal imaging study also provides in vivo evidence that zonisamide distinctly has neuroprotective potentiation in patients, helping ameliorate attentional decline.

### Supplementary Information

Supplementary Information.

Supplementary Figure 1.

Supplementary Figure 2.

Supplementary Figure 3.

Supplementary Table 1.

Supplementary Table 2.

## Data Availability

The data that support the findings of this study are available from the corresponding author upon reason-able request.

## References

[1] Müller CM (2005). Staging of sporadic Parkinson disease-related alpha-synuclein pathology: Inter- and intra-rater reliability. J. Neuropathol. Exp. Neurol..

[2] Zgaljardic DJ, Borod JC, Foldi NS, Mattis P (2003). A review of the cognitive and behavioral sequelae of Parkinson’s disease: Relationship to frontostriatal circuitry. Cogn. Behav. Neurol..

[3] Lai TT, Kim YJ, Ma HI, Kim YE (2022). Evidence of inflammation in Parkinson’s disease and its contribution to synucleinopathy. J. Mov. Disord..

[4] Terada T (2016). Extrastriatal spreading of microglial activation in Parkinson’s disease: A positron emission tomography study. Ann. Nucl. Med..

[5] Kondo T (2005). Levodopa therapy from the neuroprotection viewpoint. From a clinical outlook. J. Neurol..

[6] Murata M, Horiuchi E, Kanazawa I (2001). Zonisamide has beneficial effects on Parkinson’s disease patients. Neurosci. Res..

[7] Yokoyama H (2010). Therapeutic effect of a novel anti-parkinsonian agent zonisamide against MPTP (1-methyl-4-phenyl-1,2,3,6- tetrahydropyridine) neurotoxicity in mice. Metab. Brain Dis..

[8] Tada S (2022). Zonisamide ameliorates microglial mitochondriopathy in Parkinson’s disease models. Brain Sci..

[9] Hossain MM (2018). The anti-parkinsonian drug zonisamide reduces neuroinflammation: Role of microglial Nav 1.6. Exp. Neurol..

[10] Yokokura M (2017). Depiction of microglial activation in aging and dementia: Positron emission tomography with [(11)C]DPA713 versus [(11)C]( R)PK11195. J. Cereb. Blood Flow Metab..

[11] Ouchi Y (1999). Alterations in binding site density of dopamine transporter in the striatum, orbitofrontal cortex, and amygdala in early Parkinson’s disease: Compartment analysis for beta-CFT binding with positron emission tomography. Ann. Neurol..

[12] Hughes AJ, Daniel SE, Kilford L, Lees AJ (1992). Accuracy of clinical diagnosis of idiopathic Parkinson’s disease: A clinico-pathological study of 100 cases. J. Neurol. Neurosurg. Psychiatry.

[13] Ebadi M, Sharma S, Shavali S, El Refaey H (2002). Neuroprotective actions of selegiline. J. Neurosci. Res..

[14] Schapira AH (2013). Pramipexole in patients with early Parkinson’s disease (PROUD): A randomised delayed-start trial. Lancet Neurol..

[15] McKeith IG (2005). Diagnosis and management of dementia with Lewy bodies: Third report of the DLB Consortium. Neurology.

[16] Litvan I (1996). Clinical research criteria for the diagnosis of progressive supranuclear palsy (Steele–Richardson–Olszewski syndrome): Report of the NINDS-SPSP international workshop. Neurology.

[17] Armstrong MJ (2013). Criteria for the diagnosis of corticobasal degeneration. Neurology.

[18] Gilman S (2008). Second consensus statement on the diagnosis of multiple system atrophy. Neurology.

[19] Goetz CG, LeWitt PA, Weidenman M (2003). Standardized training tools for the UPDRS activities of daily living scale: Newly available teaching program. Mov. Disord..

[20] Hagell P, Nilsson MH (2009). The 39-Item Parkinson’s Disease Questionnaire (PDQ-39): Is it a unidimensional construct?. Ther. Adv. Neurol. Disord..

[21] Tombaugh TN, McIntyre NJ (1992). The mini-mental state examination: A comprehensive review. J. Am. Geriatr. Soc..

[22] Cummings JL (1994). The Neuropsychiatric Inventory: Comprehensive assessment of psychopathology in dementia. Neurology.

[23] Hughes CP, Berg L, Danziger WL, Coben LA, Martin RL (1982). A new clinical scale for the staging of dementia. Br. J. Psychiatry.

[24] Takashima H (2022). In vivo illustration of altered dopaminergic and GABAergic systems in early Parkinson’s disease. Front. Neurol..

[25] Kutlu MG (2021). Dopamine release in the nucleus accumbens core signals perceived saliency. Curr. Biol..

[26] Greer SM, Trujillo AJ, Glover GH, Knutson B (2014). Control of nucleus accumbens activity with neurofeedback. NeuroImage.

[27] Fang C, Lv L, Mao S, Dong H, Liu B (2020). Cognition deficits in Parkinson’s disease: Mechanisms and treatment. Parkinson’s Dis..

[28] Ferreira SA, Romero-Ramos M (2018). Microglia response during Parkinson’s disease: Alpha-synuclein intervention. Front. Cell. Neurosci..

[29] Braak H (2003). Staging of brain pathology related to sporadic Parkinson’s disease. Neurobiol. Aging.

[30] Muddapu VR, Vijayakumar K, Ramakrishnan K, Chakravarthy VS (2022). A multi-scale computational model of levodopa-induced toxicity in Parkinson’s disease. Front. Neurosci..

[31] Arawaka S (2014). Zonisamide attenuates α-synuclein neurotoxicity by an aggregation-independent mechanism in a rat model of familial Parkinson’s disease. PLoS One.

[32] Mori A, Noda Y, Packer L (1998). The anticonvulsant zonisamide scavenges free radicals. Epilepsy Res..

[33] Condello S (2013). Protective effects of zonisamide against rotenone-induced neurotoxicity. Neurochem. Res..

[34] Yokoyama H (2010). Therapeutic effect of a novel anti-parkinsonian agent zonisamide against MPTP (1-methyl-4-phenyl-1,2,3,6-tetrahydropyridine) neurotoxicity in mice. Metab. Brain Dis..

[35] Nagano-Saito A (2008). Dopamine depletion impairs frontostriatal functional connectivity during a set-shifting task. J. Neurosci..

[36] Kato TA (2012). Minocycline modulates human social decision-making: Possible impact of microglia on personality-oriented social behaviors. PLoS One.

[37] Murakami H (2017). Effects of dopaminergic drug adjustment on executive function in different clinical stages of Parkinson’s disease. Neuropsychiatr. Dis. Treat..

[38] Lewis SJ, Slabosz A, Robbins TW, Barker RA, Owen AM (2005). Dopaminergic basis for deficits in working memory but not attentional set-shifting in Parkinson’s disease. Neuropsychologia.

[39] Murata M, Hasegawa K, Kanazawa I (2007). Zonisamide improves motor function in Parkinson disease: A randomized, double-blind study. Neurology.

[40] Murata M (2018). Adjunct zonisamide to levodopa for DLB parkinsonism: A randomized double-blind phase 2 study. Neurology.

[41] Rinne JO (1999). Usefulness of a dopamine transporter PET ligand [(18)F]beta-CFT in assessing disability in Parkinson’s disease. J. Neurol. Neurosurg. Psychiatry.

[42] Miwa H (2007). Zonisamide for the treatment of Parkinson’s disease. Expert Rev. Neurother..

[43] Kong L (2022). Zonisamide’s efficacy and safety on Parkinson’s disease and dementia with Lewy bodies: A meta-analysis and systematic review. BioMed Res. Int..

[44] Ikeda K (2018). Zonisamide cotreatment delays striatal dopamine transporter reduction in Parkinson disease: A retrospective, observational cohort study. J. Neurol. Sci..

[45] Mavridis IN, Pyrgelis ES (2022). Nucleus accumbens atrophy in Parkinson’s disease (Mavridis’ atrophy): 10 years later. Am. J. Neurodegener. Dis..

