# Supplementary material for: Neuroinflammation following anti-parkinsonian drugs in early Parkinson’s disease: a longitudinal PET study
Source: Sci Rep. 2024 Feb 27;14:4708. doi: 10.1038/s41598-024-55233-z (PMC10897150; doi:10.1038/s41598-024-55233-z)
Supplement: Supplementary file 1 — Supplementary Information. [file 41598_2024_55233_MOESM1_ESM.docx]

**Supplementary Methods**

**MRI scanning**

T1 weighted magnetic resonance imaging (MRI) scans were obtained by using the following parameters: TR = 6.0, TE =2.7, flip angle 8°, slice thickness 1.0 mm, matrices 272×272, and FOV=240 mm. The intercommissural [anterior commissure-posterior commissure (AC-PC)] line on the sagittal MRI scan of each subject’s brain was determined, which enabled us to determine the brain areas in which to establish regions of interest (ROIs). The MRI measurements and mobile PET gantry allowed us to reconstruct PET images parallel to the AC-PC line without reslicing, as described below. Thus, we were able to locate the ROIs in the target regions of the original PET images.

**PET measurement**

PET was performed, yielding 47 slices simultaneously. During the PET scan, a thermoplastic face mask was used to fix the head to the same place during the scans. The orbitomeatal line of each subject was defined by direct visual inspection of the subject and was aligned with a laser. On the other hand, we analyzed the angles between the AC-PC line and orbitomeatal line on the sagittal MRI of each subject’s brain. Then, with reference to the measures of tilt angle and spatial coordinates obtained in the MRI, the PET gantry was set parallel to the AC-PC line determined by MRI by tilting and moving the gantry for each study. After backprojection and filtering (Hanning filter, cutoff frequency 0.2 cycles per pixel), the image resolution was 2.9 × 2.9 × 3.4 mm full-width half-maximum (FWHM). The voxel of each reconstructed image measured to be 1.3×1.3×3.4 mm. A 10-min transmission scan for attenuation correction with a ^68^Ge/^68^Ga source was conducted under resting conditions. Dynamic PET scans (90-minute serial emission scan with 33 frames: 6 frames×10 seconds, 3 × 20, 6 × 60, 4 × 180, and 14 × 300) were performed after a slow bolus intravenous injection (taking 1 min) of a 5 MBq/kg dose of [^11^C]DPA713. After 2.5 hours of rest, dynamic PET scans (90-minute serial emission scan with 33 frames: 6 frames×10 seconds, 3×20, 6×60, 180×4, and 14×300) were performed after an intravenous injection of a 6 MBq/kg dose of [^11^C]CFT. No arterial sampling was performed along with the series of PET measurements

**[^11^C]DPA713** **PET imaging data processing**

The validity of this procedure has been authorized by our previous report [1] [2] [3]. Polymorphic ROIs were manually located on the bilateral frontal, temporal, parietal and occipital cortices; thalamus; striatum; cerebellar hemisphere; and brain stem on MRI scans (Supplementary Figure 1). These ROIs were then automatically transferred onto corresponding [^11^C]DPA713 BP_ND_ parametric images. We analyzed the [^11^C]DPA713 time-activity curves (TACs) of each ROI in the subjects to determine the reference (receptor-less) and target (receptor-rich) regions in the simplified reference tissue model (SRTM) analysis. A normalized input curve was first created by averaging the TACs from all the ROIs in the control group. This normalized input curve from the control group was used as the TAC for the reference region of the patients and normal controls because the desirable reference region, which is free from specific binding of [^11^C]DPA713, is not present in patients with neurodegenerative disorders. When applying the normalized input curve to the individual subjects, this curve was calibrated by adjusting the lowest TAC peak for each subject. The lowest TAC for each subject was determined by using cluster analysis as follows: small circular ROIs of 2 mm in diameter were placed fully within the ROIs already determined on the MRI scan, and the TAC with the lowest peak from each TAC of all of the small ROIs (2x2x1.7) was obtained. Then, the normalized input curve adjusting each subject's lowest TAC peak was determined as the TAC in the reference region. This procedure excluded TACs from the white matter and cerebrospinal fluid space. The thalamus of each subject was chosen as a target region. Using the TAC of the target and reference regions, the BP_ND_ parametric images of [^11^C]DPA713 were generated based on SRTM. The extra cerebral structures were then masked by demarcating cerebral regions on brain MRI.

**[^11^C]CFT PET imaging data processing**

The polymorphic ROIs located manually in the bilateral caudate, putamen and cerebellum by using MRI data (Supplementary Figure 1) were automatically transferred onto corresponding [^11^C]CFT images reconstructed from 70 to 90 min after injection. The standard uptake value in the striatum was divided by the standard uptake value of the cerebellum in the same subject and was expressed as the SUVR image [4].

**Analysis of zonisamide in plasma**

Plasma samples were assayed for zonisamide determination using a HPLC-UV method. Plasma sample with the internal standard (N,N-dimethylzonisamide) was applied to solid-phase extraction column (OASIS HLB, Waters Corporation, Japan), rinsed with distilled water and distilled water/acetonitrile (9/1, v/v) in this order, and extracted with distilled water/acetonitrile (1/3, v/v). The extraction was dried up in vacuo and dissolved with methanol. And then, the sample was applied to the HPLC-UV system. The conditions of HPLC was the followings; HPLC system, Shimadzu Prominence (Shimadzu Corp, Japan); HPLC column, YMC-Pack ODS-A (YMC Co., Ltd., Japan); Mobile phase, 0.5% formic acid/acetonitrile = 67/33 (isocratic condition); and UV wavelength, 285 nm.

**REFERENCES**

1. Yokokura M, Terada T, Bunai T, Nakaizumi K, Takebayashi K, Iwata Y, Yoshikawa E, Futatsubashi M, Suzuki K, Mori N, Ouchi Y (2017) Depiction of microglial activation in aging and dementia: Positron emission tomography with [(11)C]DPA713 versus [(11)C]( R)PK11195. Journal of cerebral blood flow and metabolism : official journal of the International Society of Cerebral Blood Flow and Metabolism 37:877-889. doi: 10.1177/0271678X16646788

2. Terada T, Yokokura M, Yoshikawa E, Futatsubashi M, Kono S, Konishi T, Miyajima H, Hashizume T, Ouchi Y (2016) Extrastriatal spreading of microglial activation in Parkinson's disease: a positron emission tomography study. Annals of nuclear medicine 30:579-587. doi: 10.1007/s12149-016-1099-2

3. Terada T, Yokokura M, Obi T, Bunai T, Yoshikawa E, Ando I, Shimada H, Suhara T, Higuchi M, Ouchi Y (2019) In vivo direct relation of tau pathology with neuroinflammation in early Alzheimer's disease. Journal of neurology 266:2186-2196. doi: 10.1007/s00415-019-09400-2

4. Takashima H, Terada T, Bunai T, Matsudaira T, Obi T, Ouchi Y (2022) In vivo Illustration of Altered Dopaminergic and GABAergic Systems in Early Parkinson's Disease. Frontiers in neurology 13:880407. doi: 10.3389/fneur.2022.880407
