# Supplementary material for: Neuroinflammation following anti-parkinsonian drugs in early Parkinson’s disease: a longitudinal PET study
Source: Sci Rep. 2024 Feb 27;14:4708. doi: 10.1038/s41598-024-55233-z (PMC10897150; doi:10.1038/s41598-024-55233-z)
Supplement: Supplementary file 2 — Supplementary Figure 1. [file 41598_2024_55233_MOESM2_ESM.pptx]

## Slide 1
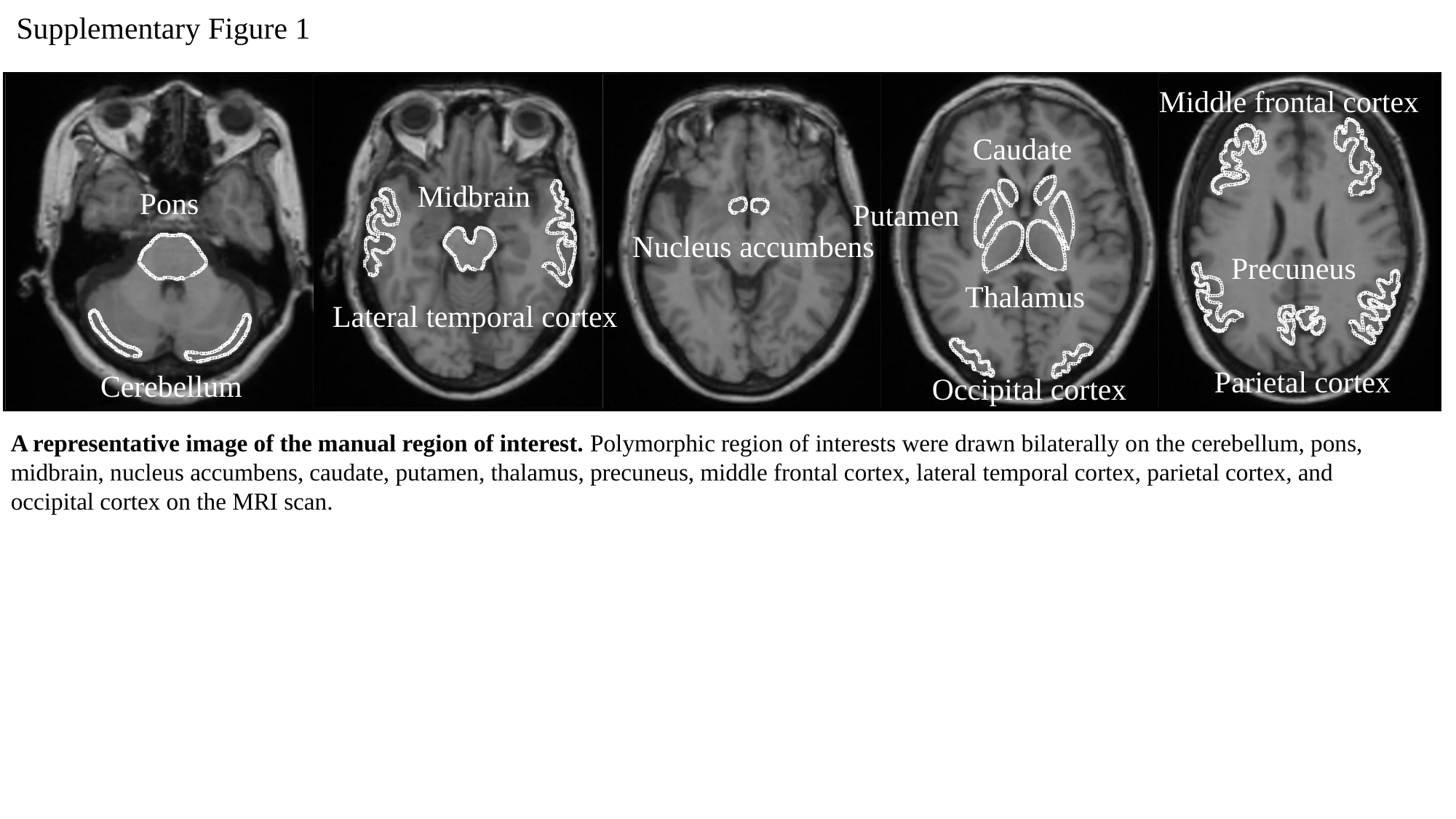

Supplementary Figure 1
Middle frontal cortex
Caudate
Midbrain
Pons
Putamen
Nucleus accumbens
Precuneus
Thalamus
Lateral temporal cortex
Parietal cortex
Cerebellum
Occipital cortex
A representative image of the manual region of interest. Polymorphic region of interests were drawn bilaterally on the cerebellum, pons, midbrain, nucleus accumbens, caudate, putamen, thalamus, precuneus, middle frontal cortex, lateral temporal cortex, parietal cortex, and occipital cortex on the MRI scan.
