# Supplementary material for: Neuroinflammation following anti-parkinsonian drugs in early Parkinson’s disease: a longitudinal PET study
Source: Sci Rep. 2024 Feb 27;14:4708. doi: 10.1038/s41598-024-55233-z (PMC10897150; doi:10.1038/s41598-024-55233-z)
Supplement: Supplementary file 3 — Supplementary Figure 2. [file 41598_2024_55233_MOESM3_ESM.pptx]

## Slide 1
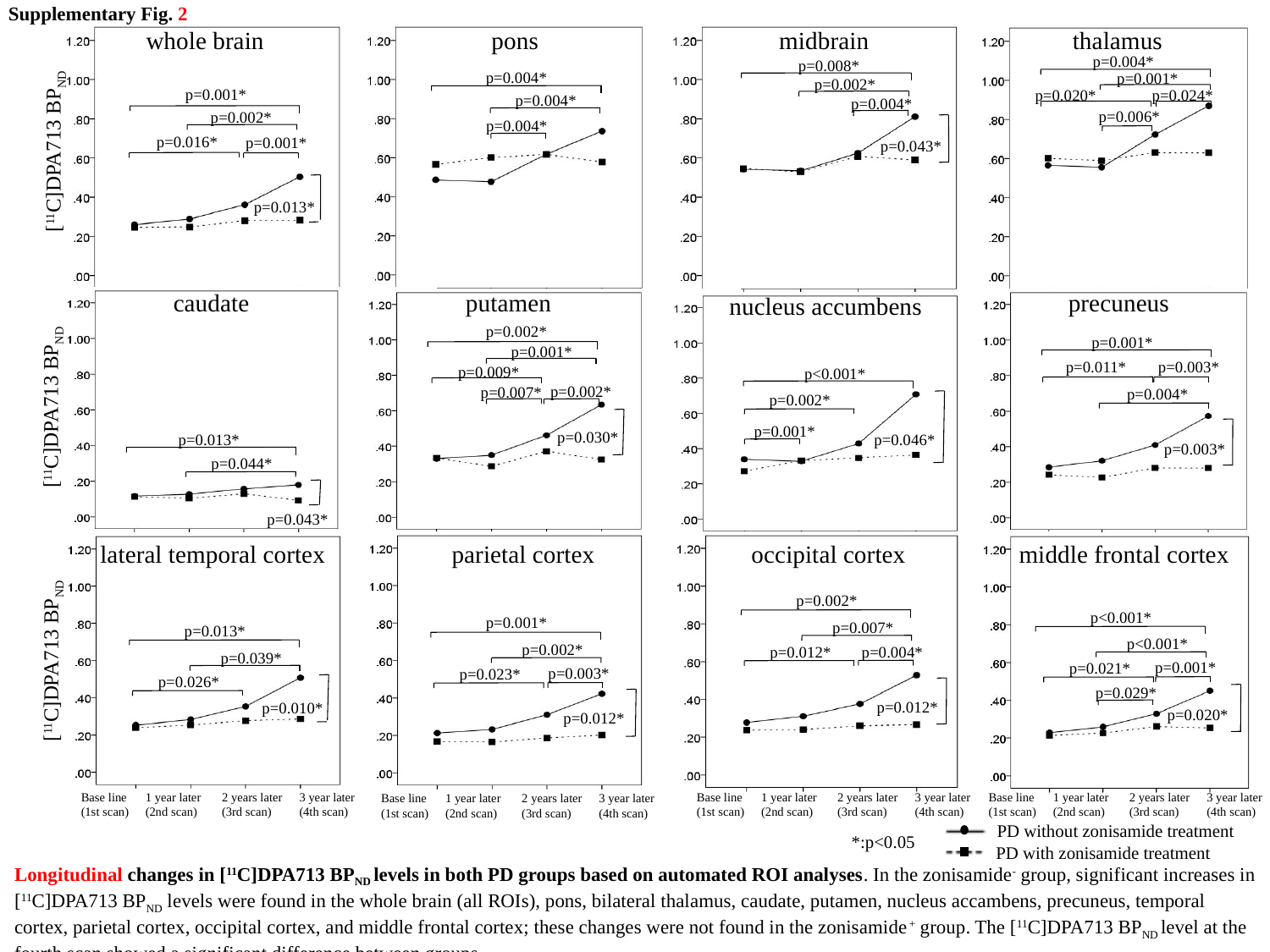

Supplementary Fig. 2
whole brain
pons
midbrain
thalamus
p=0.004*
p=0.008*
p=0.004*
p=0.001*
p=0.002*
p=0.001*
p=0.020*
p=0.024*
p=0.004*
p=0.004*
p=0.006*
p=0.002*
p=0.004*
p=0.016*
p=0.001*
[11C]DPA713 BPND
p=0.043*
p=0.013*
caudate
putamen
precuneus
nucleus accumbens
p=0.002*
p=0.001*
p=0.001*
p=0.011*
p=0.003*
p=0.009*
p<0.001*
p=0.002*
p=0.007*
p=0.004*
p=0.002*
[11C]DPA713 BPND
p=0.001*
p=0.030*
p=0.046*
p=0.013*
p=0.003*
p=0.044*
p=0.043*
lateral temporal cortex
parietal cortex
occipital cortex
middle frontal cortex
p=0.002*
p<0.001*
p=0.001*
p=0.007*
p=0.013*
p<0.001*
p=0.002*
p=0.012*
p=0.004*
[11C]DPA713 BPND
p=0.039*
p=0.001*
p=0.021*
p=0.003*
p=0.023*
p=0.026*
p=0.029*
p=0.012*
p=0.010*
p=0.020*
p=0.012*
Base line
(1st scan)
1 year later
(2nd scan)
2 years later
(3rd scan)
3 year later
(4th scan)
Base line
(1st scan)
1 year later
(2nd scan)
2 years later
(3rd scan)
3 year later
(4th scan)
Base line
(1st scan)
1 year later
(2nd scan)
2 years later
(3rd scan)
3 year later
(4th scan)
Base line
(1st scan)
1 year later
(2nd scan)
2 years later
(3rd scan)
3 year later
(4th scan)
PD without zonisamide treatment
*:p<0.05
PD with zonisamide treatment
Longitudinal changes in [11C]DPA713 BPND levels in both PD groups based on automated ROI analyses. In the zonisamide- group, significant increases in [11C]DPA713 BPND levels were found in the whole brain (all ROIs), pons, bilateral thalamus, caudate, putamen, nucleus accambens, precuneus, temporal cortex, parietal cortex, occipital cortex, and middle frontal cortex; these changes were not found in the zonisamide+ group. The [11C]DPA713 BPND level at the fourth scan showed a significant difference between groups.
