# Supplementary material for: Neuroinflammation following anti-parkinsonian drugs in early Parkinson’s disease: a longitudinal PET study
Source: Sci Rep. 2024 Feb 27;14:4708. doi: 10.1038/s41598-024-55233-z (PMC10897150; doi:10.1038/s41598-024-55233-z)
Supplement: Supplementary file 4 — Supplementary Figure 3. [file 41598_2024_55233_MOESM4_ESM.pptx]

## Slide 1
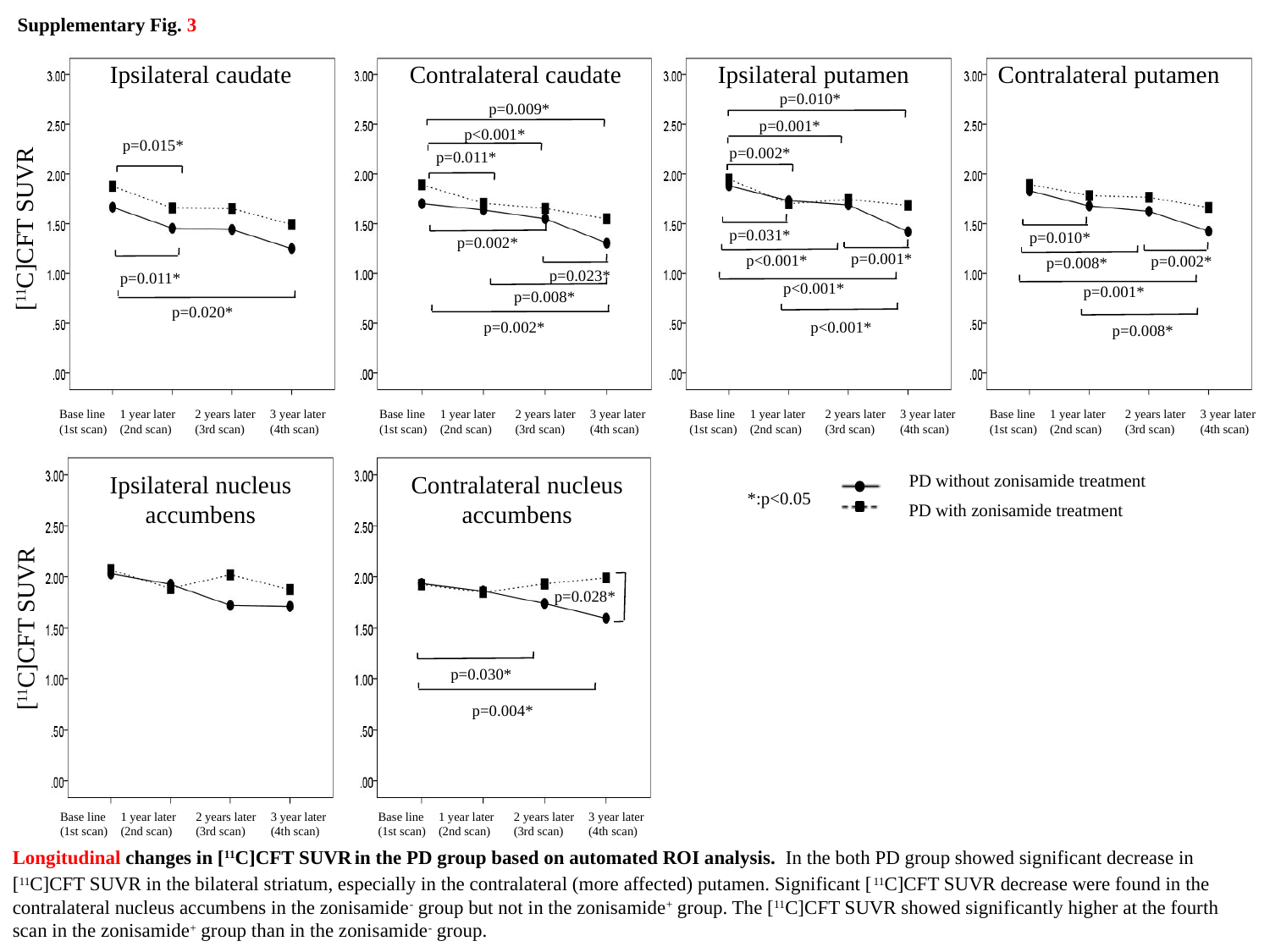

Supplementary Fig. 3
Ipsilateral caudate
Contralateral caudate
Ipsilateral putamen
Contralateral putamen
p=0.010*
p=0.009*
p=0.001*
p<0.001*
p=0.015*
p=0.002*
p=0.011*
[11C]CFT SUVR
p=0.031*
p=0.010*
p=0.002*
p=0.001*
p<0.001*
p=0.002*
p=0.008*
p=0.023*
p=0.011*
p<0.001*
p=0.001*
p=0.008*
p=0.020*
p=0.002*
p<0.001*
p=0.008*
Base line
(1st scan)
1 year later
(2nd scan)
2 years later
(3rd scan)
3 year later
(4th scan)
Base line
(1st scan)
1 year later
(2nd scan)
2 years later
(3rd scan)
3 year later
(4th scan)
Base line
(1st scan)
1 year later
(2nd scan)
2 years later
(3rd scan)
3 year later
(4th scan)
Base line
(1st scan)
1 year later
(2nd scan)
2 years later
(3rd scan)
3 year later
(4th scan)
Ipsilateral nucleus accumbens
Contralateral nucleus accumbens
PD without zonisamide treatment
*:p<0.05
PD with zonisamide treatment
p=0.028*
[11C]CFT SUVR
p=0.030*
p=0.004*
Base line
(1st scan)
1 year later
(2nd scan)
2 years later
(3rd scan)
3 year later
(4th scan)
Base line
(1st scan)
1 year later
(2nd scan)
2 years later
(3rd scan)
3 year later
(4th scan)
Longitudinal changes in [11C]CFT SUVR in the PD group based on automated ROI analysis. In the both PD group showed significant decrease in [11C]CFT SUVR in the bilateral striatum, especially in the contralateral (more affected) putamen. Significant [11C]CFT SUVR decrease were found in the contralateral nucleus accumbens in the zonisamide- group but not in the zonisamide+ group. The [11C]CFT SUVR showed significantly higher at the fourth scan in the zonisamide+ group than in the zonisamide- group.
