# Supplementary material for: Neuroinflammation following anti-parkinsonian drugs in early Parkinson’s disease: a longitudinal PET study
Source: Sci Rep. 2024 Feb 27;14:4708. doi: 10.1038/s41598-024-55233-z (PMC10897150; doi:10.1038/s41598-024-55233-z)
Supplement: Supplementary file 5 — Supplementary Table 1. [file 41598_2024_55233_MOESM5_ESM.docx]

Supplementary Table 1A．Levels of [^11^C]DPA713 BPND in the Parkinson’s disease group (manual ROI analysis)

|  | Parkinson’s disease group | | | |
| --- | --- | --- | --- | --- |
|  | Subgroup with Zonisamide treatment / Subgroup without Zonisamide treatment | | | |
|  | 1st scan | 2nd scan | 3rd scan | 4th scan |
| Whole brain | 0.40±0.11 / 0.39±0.21 | 0.42±0.11 / 0.43±0.22 | 0.44±0.09 / 0.54±0.23 | 0.46±0.09 / 0.72±0.20 |
| Pons | 0.79±0.21 / 0.75±.32 | 0.88±0.25 / 0.73±0.28 | 0.79±0.13 / 0.85±0.18 | 0.88±0.16 / 1.08±0.26 |
| Midbrain | 0.46±0.14 / 0.51±0.23 | 0.46±0.13 / 0.51±0.20 | 0.51±0.04 / 0.59±0.16 | 0.50±0.14 / 0.72±0.28 |
| Thalamus | 0.66±0.19 / 0.62±0.31 | 0.68±0.19 / 0.62±0.28 | 0.72±0.17 / 0.83±0.36 | 0.71±0.12 / 1.01±0.24 |
| Caudate | 0.09±0.01 / 0.14±.11 | 0.11±0.05 / 0.19±0.20 | 0.13±0.04 / 0.28±0.23 | 0.13±0.02 / 0.33±0.14 |
| Putamen | 0.28±0.09 / 0.28±0.20 | 0.25±0.10 / 0.35±0.24 | 0.32±0.11 / 0.45±0.29 | 0.32±0.10 / 0.60±0.26 |
| Nuclear accumbens | 0.33±0.17 / 0.39±0.24 | 0.43±0.23 / 0.40±0.20 | 0.35±0.11 / 0.51±0.30 | 0.36±0.15 / 0.83±0.37 |
| Precuneus | 0.38±0.15 / 0.46±0.24 | 0.41±0.10 / 0.55±0.30 | 0.45±0.16 / 0.63±0.25 | 0.48±0.11 / 0.82±0.17 |
| Lateral temporal cortex | 0.22±0.09 / 0.27±0.16 | 0.27±0.13 / 0.27±0.15 | 0.29±0.07 / 0.38±0.18 | 0.33±0.09 / 0.61±0.17 |
| Parietal cortex | 0.27±0.11 / 0.29±0.17 | 0.27±0.10 / 0.33±0.21 | 0.31±0.08 / 0.45±0.23 | 033±0.07 / 0.63±0.19 |
| Occipital cortex | 0.37±0.10 / 0.39±0.23 | 0.33±0.10 / 0.34±0.17 | 0.37±0.10 / 0.48±0.23 | 0.37±0.07 / 0.73±0.20 |
| Middle frontal cortex | 0.32±0.10 / 0.29±0.18 | 0.35±0.13 / 0.37±0.22 | 0.41±0.15 / 0.49±0.24 | 0.43±.10 / 0.67±0.19 |

Data are presented as mean ± SD (range).

Abbreviations: ROI, region of interest

Supplementary Table 1B．Levels of [^11^C]DPA713 BPND in the Parkinson’s disease group (automated ROI analysis)

|  | Parkinson’s disease group | | | |
| --- | --- | --- | --- | --- |
|  | Subgroup with Zonisamide treatment / Subgroup without Zonisamide treatment | | | |
|  | 1st scan | 2nd scan | 3rd scan | 4th scan |
| Whole brain | 0.25±0.06 / 0.26±0.14 | 0.25±0.07 / 0.29±0.16 | 0.28±0.06 / 0.36±0.18 | 0.28±0.06 / 0.50±0.15 |
| Pons | 0.56±0.14 / 0.49±0.20 | 0.60±0.15 / 0.48±0.20 | 0.62±0.12 / 0.62±0.20 | 0.58±0.11 / 0.74±0.20 |
| Midbrain | 0.55±0.17 / 0.54±0.23 | 0.53±0.13 / 0.53±0.22 | 0.61±0.07 / 0.62±0.19 | 0.59±0.13 / 0.81±0.17 |
| Thalamus | 0.60±0.13 / 0.56±0.28 | 0.59±0.11 / 0.55±0.25 | 0.63±0.09 / 0.72±0.31 | 0.63±0.10 / 0.87±0.24 |
| Caudate | 0.11±0.03 / 0.12±0.09 | 0.10±0.05 / 0.13±0.10 | 0.13±0.02 / 0.16±0.13 | 0.09±0.02 / 0.18±0.08 |
| Putamen | 0.33±0.10 / 0.33±0.20 | 0.29±.009 / 0.35±0.22 | 0.37±0.12 / 0.46±0.24 | 0.33±0.10 / 0.63±0.25 |
| Nuclear accumbens | 0.27±0.12 / 0.34±0.27 | 0.33±0.14 / 0.33±0.19 | 0.35±0.14 / 0.43±0.23 | 0.36±0.09 / 0.71±0.32 |
| Precuneus | 0.24±0.07 / 0.28±0.15 | 0.23±0.06 / 0.32±0.16 | 0.28±0.06 / 0.41±0.18 | 0.28±0.03 / 0.57±0.16 |
| Lateral temporal cortex | 0.24±0.07 / 0.25±0.14 | 0.25±0.08 / 0.28±0.16 | 0.28±0.06 / 0.35±0.17 | 0.29±0.07 / 0.51±0.15 |
| Parietal cortex | 0.17±0.06 / 0.21±0.12 | 0.16±0.05 / 0.23±0.14 | 0.19±0.05 / 0.31±0.17 | 0.20±0.04 / 0.42±0.15 |
| Occipital cortex | 0.24±0.06 / 0.28±0.14 | 0.24±0.07 / 0.31±0.16 | 0.26±0.04 / 0.38±0.15 | 0.27±0.04 / 0.53±0.13 |
| Middle frontal cortex | 0.21±0.06 / 0.23±0.13 | 0.23±0.08 / 0.26±0.14 | 0.26±0.07 / 0.33±0.17 | 0.25±0.06 / 0.45±0.14 |

Data are presented as mean ± SD (range).

Abbreviations: ROI, region of interest

Supplementary Table 1C．Demographic and clinical characteristics of the Parkinson's disease (PD) group

|  | Parkinson’s disease group | | | |
| --- | --- | --- | --- | --- |
|  | Subgroup with Zonisamide treatment / Subgroup without Zonisamide treatment | | | |
|  | 1st scan | 2nd scan | 3rd scan | 4th scan |
| The amount of change from the baseline |  |  |  |  |
| Hoehn & Yahr stage | 0.00±0.00 / 0.00±0.00 | 0.00±0.00 / 0.33±0.82 | 0.40±0.89 / -0.17±0.41 | 0.20±0.45 / 0.00±0.63 |
| Unified PD Rating Scale part 1 | 0.00±0.00 / 0.00±0.00 | 0.20±1.30 / -0.50±1.76 | -0.80±1.92 / -0.67±1.75 | -0.80±1.64 / -0.50±1.38 |
| Unified PD Rating Scale part 2 | 0.00±0.00 / 0.00±0.00 | -0.20±1.30 / -0.33±1.21 | 1.80±3.19 / -1.50±2.66 | -0.40±4.77 / -1.50±2.07 |
| Unified PD Rating Scale part 3 | 0.00±0.00 / 0.00±0.00 | 0.00±4.36 / -1.50±1.76 | 1.40±3.78 / -0.50±4.14 | -4.40±9.24 / -0.67±3.50 |
| PD Questionnaire 39 summary index | 0.00±0.00 / 0.00±0.00 | 3.08±6.6 / -6.3±8.8 | 2.56±3.0 / 0.00±10.9 | 1.41±9.1 / -3.3±8.4 |
| Toal Mini-Mental State Examination | 0.00±0.00 / 0.00±0.00 | 0.80±2.77 / 0.17±2.32 | 2.60±1.95 / 1.17±2.48 | 2.80±1.92 / 1.17±2.64 |
| Seven series subscore of  Mini-Mental State Examination | 0.00±0.00 / 0.00±0.00 | 2.80±1.79 / 0.33±2.2.5 | 3.20±1.79 / 0.67±1.97 | 3.20±1.79 / 0.67±1.86 |
| Neuropsychiatric Inventory | 0.00±0.00 / 0.00±0.00 | -0.40±3.71 / -2.17±5.31 | 1.00±5.79 / -1.83±5.53 | -3.60±5.55 / -2.17±5.31 |
| Levodopa daily dose (mg/day) | 350.0±190.4 / 333.3±227.3 | 360.0±185.1 / 350.0±209.8 | 430.0±171.8 / 350.0±209.8 | 510.0±185.1 / 383.3±183.5 |
| Levodopa equivalent daily dose (mg/day) | 350.0±190.4 / 341.7±217.8 | 360.0±185.1 / 382.4±232.0 | 469.8±171.4 / 387.9±234.3 | 602.8±262.8 / 423.3±212.7 |
| Zonisamide daily dose (mg/day) | 0.00±0.00 / 0.00±0.00 | 25.0±0.00 / 0.00±0.00 | 30.0±11.2 / 0.00±0.00 | 35.0±13.7 / 0.00±0.00 |
| Zonisamide concentration (μg/ml) | 0.00±0.00 / 0.00±0.00 | 1.89±0.95 / 0.00±0.00 | 2.26±1.38 / 0.00±0.00 | 2.76±1.27 / 0.00±0.00 |

Data are presented as mean ± SD (range).

Supplementary Table 1D．Levels of [^11^C]CFT standard uptake value ratio in the Parkinson’s disease group (manual ROI analysis)

|  | Parkinson’s disease group | | | |
| --- | --- | --- | --- | --- |
|  | Subgroup with Zonisamide treatment / Subgroup without Zonisamide treatment | | | |
|  | 1st scan | 2nd scan | 3rd scan | 4th scan |
| Ipsilateral Caudate | 3.12±0.35 / 2.96±0.35 | 3.02±0.37 / 2.64±0.24 | 2.92±0.34 / 2.51±0.45 | 2.45±0.20 / 2.01±0.41 |
| Contralateral Caudate | 2.90±0.37 / 2.58±0.26 | 2.75±0.35 / 2.45±0.20 | 2.42±0.40 / 2.32±0.29 | 2.32±0.18 / 2.01±0.18 |
| Ipsilateral Putamen | 2.33±0.33 / 2.26±0.22 | 2.10±0.26 / 2.05±0.30 | 2.01±0.25 / 1.96±0.29 | 1.92±0.25 / 1.58±0.29 |
| Contralateral Putamen | 1.95±0.33 / 1.94±0.28 | 1.91±0.31 / 1.78±0.27 | 1.86±0.31 / 1.75±0.30 | 1.68±0.20 / 1.44±0.19 |
| Ipsilateral Accumbens | 2.82±0.37 / 2.80±0.52 | 2.57±0.35 / 2.47±0.34 | 2.78±0.66 / 2.44±0.54 | 2.44±0.20 / 2.18±0.35 |
| Contralateral Accumbens | 2.83±0.33 / 2.38±0.38 | 2.50±0.35 / 2.56±0.27 | 2.60±0.42 / 2.32±0.32 | 2.63±0.35 / 2.01±0.30 |

Data are presented as mean ± SD (range).

Abbreviations: ROI, region of interest

Supplementary Table 1E．Levels of [^11^C]CFT standard uptake value ratio in the Parkinson’s disease group (automated ROI analysis)

|  | Parkinson’s disease group | | | |
| --- | --- | --- | --- | --- |
|  | Subgroup with Zonisamide treatment / Subgroup without Zonisamide treatment | | | |
|  | 1st scan | 2nd scan | 3rd scan | 4th scan |
| Ipsilateral Caudate | 1.88±0.37 / 1.67±0.28 | 1.66±0.44 / 1.45±0.25 | 1.65±0.48 / 1.44±0.25 | 1.49±0.34 / 1.25±0.27 |
| Contralateral Caudate | 1.89±0.27 / 1.70±0.12 | 1.71±0.25 / 1.64±0.10 | 1.66±0.23 / 1.55±0.09 | 1.55±0.19 / 1.31±0.13 |
| Ipsilateral Putamen | 1.95±0.23 / 1.88±0.14 | 1.70±0.15 / 1.73±0.16 | 1.75±0.16 / 1.69±0.17 | 1.69±0.19 / 1.42±0.21 |
| Contralateral Putamen | 1.90±0.24 / 1.83±0.16 | 1.78±0.23 / 1.68±0.14 | 1.77±0.16 / 1.63±0.19 | 1.66±0.18 / 1.43±0.17 |
| Ipsilateral Accumbens | 2.07±0.20 / 2.03±0.34 | 1.89±0.34 / 1.93±0.22 | 2.02±0.20 / 1.72±0.33 | 1.87±0.19 / 1.71±.30 |
| Contralateral Accumbens | 1.92±0.33 / 1.93±0.27 | 1.85±0.19 / 1.86±0.21 | 1.93±0.22 / 1.74±0.31 | 1.99±0.27 / 1.59±0.23 |

Data are presented as mean ± SD (range).

Abbreviations: ROI, region of interest
