# Supplementary material for: Neuroinflammation following anti-parkinsonian drugs in early Parkinson’s disease: a longitudinal PET study
Source: Sci Rep. 2024 Feb 27;14:4708. doi: 10.1038/s41598-024-55233-z (PMC10897150; doi:10.1038/s41598-024-55233-z)
Supplement: Supplementary file 6 — Supplementary Table 2. [file 41598_2024_55233_MOESM6_ESM.docx]

Supplementary Table 2．Course of medication in the patients with Parkinson's disease (PD) included in this 3-year analysis

|  | PD group | | | *P* value |
| --- | --- | --- | --- | --- |
|  | Total | Subgroup with zonisamide treatment | Subgroup without zonisamide treatment |  |
| Patients receiving COMT inhibitor/amantadine/MAO-B inhibitor (number) |  |  |  |  |
| 1st scan (base line) | 0/1*/0 (N = 16) | 0/0/0 (N=8) | 0/1*/0 (N=8) | n.s. |
| 2nd scan (1year later) | 0/1/1 (N = 16) | 0/0/0 (N=8) | 0/1/1 (N=8) | n.s. |
| 3rd scan (2years later) | 4/2/1 (N=14) | 1/1/0 (N=6) | 3/1/1 (N=8) | n.s. |
| 4th scan (3years later) | 3/2/2 (N=11) | 1/1/1 (N=5) | 2/1/1 (N=6) | n.s. |
| Levodopa equivalent daily dose (mg/day) |  |  |  |  |
| 1st scan (base line) | 337.5±161.8 | 331.3±146.2 | 343.8±186.0 | n.s. |
| 2nd scan (1year later) | 365.3±167.2 | 343.8±142.5 | 386.8±196.2 | n.s. |
| 3rd scan (2years later) | 476.8±263.0 | 458.2±156.0 | 490.8±332.6 | n.s. |
| 4th scan (3years later) | 504.9±243.0 | 602.8±262.9 | 423.3±212.7 | n.s. |

Data are presented as the mean ± SD (range).

*One patient received amantadine at entry.

After a one-year fixed protocol period, a small amount of levodopa/DCI and other anti-parkinsonian drugs were added due to exacerbation of parkinsonism. In the PD with ZNS therapy group, the levodopa/DCI dose was increased in five patients, amantadine was administered to one patient, COMT inhibitor (entacapone) was administered to one patient, and MAO-B inhibitor (rasagiline) was administered to one patient. In the PD without ZNS therapy group, the levodopa/DCI dose was increased in four patients, COMT inhibitor (entacapone) was administered to three patients, and MAO-B inhibitor (selegiline) was administered to one patient.

Abbreviations: ZNS, zonisamide; COMT, catechol-O-methyltransferase; MAO-B, monoamine oxidase-B

Conversion factor for the levodopa equivalent daily dose: entacapone, L-dopa×0.33; amantadine, ×1; selegiline, ×10; rasagiline, ×100
